# A Critical Role for the Hippocampus in the Valuation of Imagined Outcomes

**DOI:** 10.1371/journal.pbio.1001684

**Published:** 2013-10-22

**Authors:** Maël Lebreton, Maxime Bertoux, Claire Boutet, Stéphane Lehericy, Bruno Dubois, Philippe Fossati, Mathias Pessiglione

**Affiliations:** 1 Motivation, Brain and Behavior (MBB) Team, Institut du Cerveau et de la Moelle Epinière (ICM), Paris, France; 2 Service de Neuroradiologie, Hôpital Pitie-Salpetriere, Centre de NeuroImagerie de Recherche (CENIR), Institut du Cerveau et de la Moelle épinière (ICM), Paris, France; 3 INSERM UMRS 975, CNRS UMR 7225, Université Pierre et Marie Curie (UPMC – Paris 6), Paris, France; 4 Institut de la Mémoire et de la Maladie d'Alzheimer, Hôpital Pitié-Salpêtrière, Paris, France; 5 Centre Emotion, CNRS USR 3246, Hôpital Pitié-Salpêtrière, Paris, France; University of Oxford, United Kingdom

## Abstract

Many choice situations require imagining potential outcomes, a capacity that was shown to involve memory brain regions such as the hippocampus. We reasoned that the quality of hippocampus-mediated simulation might therefore condition the subjective value assigned to imagined outcomes. We developed a novel paradigm to assess the impact of hippocampus structure and function on the propensity to favor imagined outcomes in the context of intertemporal choices. The ecological condition opposed immediate options presented as pictures (hence directly observable) to delayed options presented as texts (hence requiring mental stimulation). To avoid confounding simulation process with delay discounting, we compared this ecological condition to control conditions using the same temporal labels while keeping constant the presentation mode. Behavioral data showed that participants who imagined future options with greater details rated them as more likeable. Functional MRI data confirmed that hippocampus activity could account for subjects assigning higher values to simulated options. Structural MRI data suggested that grey matter density was a significant predictor of hippocampus activation, and therefore of the propensity to favor simulated options. Conversely, patients with hippocampus atrophy due to Alzheimer's disease, but not patients with Fronto-Temporal Dementia, were less inclined to favor options that required mental simulation. We conclude that hippocampus-mediated simulation plays a critical role in providing the motivation to pursue goals that are not present to our senses.

## Abstract 2

Converging evidence from neuroimaging and clinical data demonstrates the important involvement of the hippocampus in finding the motivation to pursue goals that we need to imagine because they are not within sight.

## Author Summary

Economic theory assumes that we assign some sort of value to options that are presented to us in order to choose between them. In neuroscience, evidence suggests that memory brain regions, such as the hippocampus, are involved in imagining novel situations. We therefore hypothesized that the hippocampus might be critical for evaluating outcomes that we need to imagine. This is typically the case in intertemporal choices, where immediate rewards are considered against future gratifications (e.g., a beer now or a bottle of champagne a week from now). Previous investigations have implicated the dorsal prefrontal cortex brain region in resisting immediate rewards. Here we manipulated the mode of presentation (text or picture), such that options were represented either in simulation or in perception systems. Functional neuroimaging data confirmed that hippocampal activity lends a preference to choosing simulated options (irrespective of time), whereas dorsal prefrontal cortex brain activity supports the preference for delayed options (irrespective of presentation mode). Structural neuroimaging in healthy subjects and in patients with brain atrophy, due to Alzheimer's disease (with hippocampal damage) or Fronto-Temporal Dementia (with damage to the prefrontal cortex), further demonstrated the critical implication of the hippocampus. Individuals with higher neuronal density in the hippocampus, but not in the dorsal prefrontal cortex, were more likely to choose future rewards that have to be mentally simulated.

## Introduction

Would you prefer a can of beer today or a bottle of champagne in one week? Intertemporal choices, involving trade-offs between short-term and long-term outcomes, are pervasive in everyday life. The propensity to favor short-term pleasures defines a form of impulsivity that may have dramatic consequences on professional careers or family relationships. How can some people resist the attraction of short-term pleasures and pursue long-term goals, while others easily succumb and compromise their ultimate expectations? This issue has been tackled in the recent years using functional neuroimaging techniques to explore neural activity during intertemporal choices [1],[2]. Most studies implemented binary choices derived from behavioral economics paradigms, in which subjects have to choose between smaller-sooner and bigger-later monetary payoffs. Choice data could be fitted with a hyperbolic decay function, which characterizes how monetary payoffs are discounted over time and hence captures individual impulsivity [3]–[5]. Neural data suggested that recruitment of the dorsal prefrontal cortex is crucial to resist the attraction of immediate rewards, which is mediated by ventral prefronto-striatal circuits [6]–[8].

However, paradigms employing monetary rewards may miss some essential processes that crucially determine intertemporal choices in everyday life. A long time ago, Aristotle pointed out that “when some desirable object is not actually present to our senses, exerting its pull on us directly, our motivation to strive to obtain it is driven by our awareness of its (memory or fantasy) image” [9]. Along the same lines, some more recent authors suggested that imagining future situations might help in providing a motivation that counters the attraction of immediate pleasures [10]–[12]. Imagining future situations involves recomposing elements stored in episodic memory and hence recruiting the medial temporal lobe (MTL) regions. Indeed, these regions, with the hippocampus as a key component, are thought to be implicated in both recalling past episodes and imagining future episodes [13],[14]. This idea was principally suggested by the observation of patients with MTL damage, who exhibit parallel impairment in episodic memory and future simulation [15]–[18]. The MTL general function has consequently been conceptualized as episodic thinking or mental time travelling [11],[19]–[22]. Therefore, favoring long-term goals should involve not only the dorsal prefrontal cortex but also the medial temporal regions, as subjects engage in imagining future episodes.

The aim of the present study was to uncover the role of the hippocampus in the conflict defined by Aristotle between temptations that strike our senses and fictions that we have to generate. It has been argued that such conflict between tangible and simulated options represents the most typical case of intertemporal choice we have to make in ecological situations [12]. We therefore extended previous intertemporal choice paradigms by showing concrete options (food, culture, and sport items) with two modes of presentation: some options were accompanied with pictures and thus immediately observable through vision, whereas other options were only described textually and thus required mental simulation. We first verified in a pilot behavioral study that participants assign higher values (likeability ratings) to the options imagined with more details. Then we used functional MRI to analyze neural activity elicited by option presentation and choice response, which were separated in time. Our prediction was that in ecological situations, which opposed simulated to observable rewards, hippocampus activity would be associated with higher value assigned to the delayed option. This was not expected in control conditions using the same difference in time (immediate versus delayed options), but no difference in the presentation mode. Thus, hippocampus activation would explain a significant part of intersubject variability in the propensity to favor imagined outcomes, irrespective of delay.

To complete our demonstration, we intended to establish a critical link with hippocampus anatomical structure, and not only a correlation with functional activation. First, we regressed the degree of impulsivity exhibited by our healthy participants in the ecological choices against grey matter density measured by structural MRI, using voxel-based morphometry (VBM) analysis. The prediction was that subjects preferring imagined outcomes would show increased grey matter density in the hippocampus. Second, we tested on the same intertemporal choice task patients with Alzheimer's disease (AD), who represent the prototypical case of episodic memory impairment due to hippocampus degeneration [23],[24]. As controls we included elderly healthy subjects and patients with moderate behavioral variant of fronto-temporal dementia (bvFTD), another degenerative disease that preferentially affects the prefrontal cortex (PFC) [25],[26]. The prediction was that AD patients should make more impulsive choices in the ecological situation, when delayed options have to be simulated and hence need the hippocampus to attain higher values, relative to control groups and conditions.

## Results

**Figure 1:**
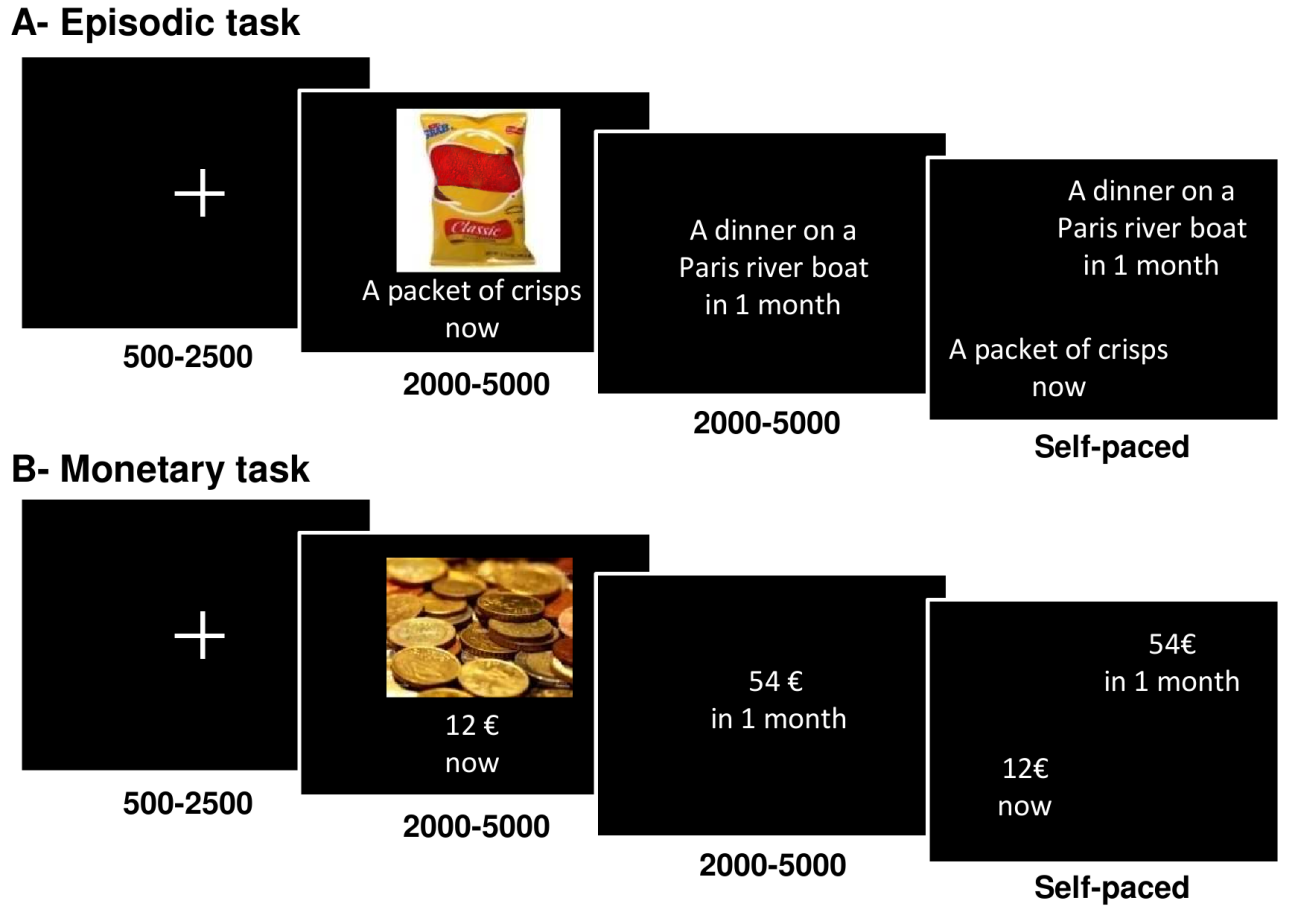
Intertemporal choice tasks. Successive screens displayed in one trial are shown from left to right with durations in ms. Subjects first watched the two options and then indicated their preference. “Simulated” options were only described textually, whereas “observed” options were additionally illustrated with a picture. Choices were given by pressing one of two buttons with the left or right hand. (A) Episodic choice task: the two options were food, culture, or sport events. (B) Monetary choice task: the two options were financial payoffs. For both tasks, the figure only illustrates the ecological condition, in which the immediate option is observed and the delayed option is simulated. The two tasks also included control conditions, with simulated immediate options and observed delayed options. The presentation order of immediate and delayed options was counterbalanced across trials.

We developed two intertemporal choice tasks (Figure 1). A first control “monetary task” was based on classical delay discounting paradigms used in neuroeconomics that oppose a low immediate payoff to a higher delayed payoff [1],[2],[6],[27]–[29]. The main “episodic task” was based on more recent neuroeconomic paradigms [30],[31] that propose less abstract options, in our case food, sport, or culture events (see Table 1 for example items). Both task performances were modeled combining hyperbolic delay discounting and a softmax decision rule. The model was primarily fed with values, which were financial payoffs for the monetary task and postscan likeability ratings for the episodic task. For every choice, the model started by discounting the value of the delayed option (by one month, one year, or ten years). Then it converted the two option values into a probability (or likelihood) of choosing the immediate versus delayed option (or impulsive versus nonimpulsive choice).

**Table 1:** Example items taken from the three domains (food, culture, and sport), ordered with increasing prices from top to bottom (all between 1 and 100 €).

Sport	Culture	Food
A free bowling session in a bar	A visit of the Ménagerie du Jardin des Plantes	A packet of crisps
A hiking session in Fontainebleau	A visit of the Palais de la Découverte	A piece of cheesecake
One hour of body massage	A 1-hour chess lesson	A glass of red wine
An initiation to Aikido practice	A guided tour of Centre Pompidou	A cup of Champagne
An indoor climbing session in Bercy	A guided tour of the Musée du Louvre	A lunch in Italian pizzeria
A rawing session on Cergy lake	A day at the Château de Versailles	A Japanese meal in front of Notre-Dame cathedral
A seat for a premier league rugby game	A salsa dancing lesson	A diner on a Paris river boat
A seat for a first league football game	A 2-h oenology lesson	A plate of seafood on the Champs-Elysées
A horse riding tour in the Bois de Vincennes	A theater play at the Comédie Française	A breakfast at the Tour Eiffel restaurant
A seat for the Rolland Garros tennis tournament final	A concert in a Paris Jazz Club	A lobster in the Tour Montparnasse restaurant

Our design did not allow comparing the impulsiveness of choices or the steepness of delay discounting between tasks, since the values were expressed in different units—that is, either in euros (in the monetary task) or in terms of likeability ratings (in the episodic task). Our main objective was to assess the role of episodic simulation in valuating delayed rewards so as to counter the attraction of immediate rewards. To this aim, we manipulated the mode of option display in both the monetary and episodic tasks. Some options were only described by a short text and hence required episodic simulation to be properly valuated, whereas other options were accompanied by a picture and hence could be valuated through direct observation (Figure 1). We then compared ecological choices, where the immediate option was observed and the delayed option simulated (Obs/Sim trials), to control choices, where both options were either observed or simulated (Obs/Obs and Sim/Sim trials). These control conditions were meant to assess the effect of delays irrespective of the presentation mode (pictures versus texts).

### Correlation of Impulsivity with Simulation Richness

**Figure 2:**
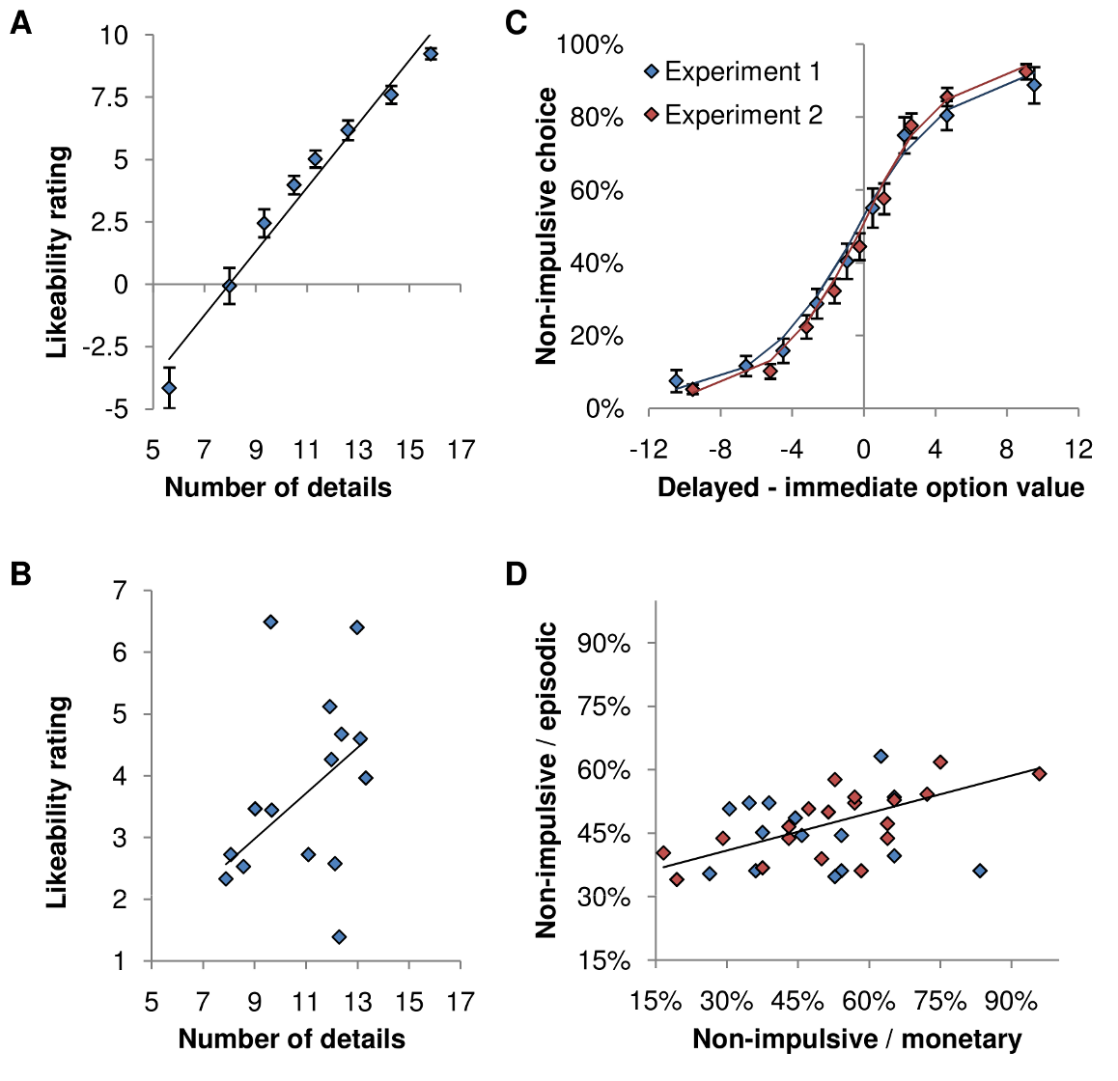
Behavioral analysis. Left: Correlation between likeability rating and simulation richness (number of details reported per option). (A) Correlation across trials (data were binned into eight data points). (B) Correlation across participants (each dot is one subject). Right: Analysis of nonimpulsive choice rate (preference for the delayed option). (C) Nonimpulsive choice rate as a function of the difference in value estimates between immediate and delayed options. Differential values were individually calculated using hyperbolic discounting for every option, then binned into nine data points and averaged across subjects. Solid lines indicate model estimates using a softmax decision rule. (D) Intersubject correlation of nonimpulsive choice rate between monetary and episodic tasks. In all scatter plots, lines represent robust regression fits. In all panels blue and red diamonds represent data from Experiments 1 and 2. Error bars indicate intersubject standard errors of the mean.

In a first behavioral pilot experiment, we verified that the quality of simulation indeed enhanced the values assigned to textually described options. Participants (*n* = 15) of Experiment 1 first performed the monetary and episodic intertemporal choice tasks and then were asked how many details they imagined when reading each Sim option. Number of details was used as a proxy for simulation richness, as was implemented in studies that established the link between episodic memory and future simulation deficits [16],[32],[33]. Simulation richness was significantly correlated across trials to subjective likeability ratings (Figure 2A, one-sample *t* test on individual robust regression coefficients, t_14_ = 8.69, *p*<0.001). Consistently, when considering ecological trials (contrasting Sim to Obs options), subjects were significantly more prone to favor the delayed option when it was simulated with higher richness (one-sample *t* test on individual robust regression coefficients, t_14_ = 6.47, *p*<0.001). The same relation between valuation and richness was observed across subjects: participants who reported having imagined more details gave higher ratings to Sim options (Figure 2B, robust regression, t_13_ = 1.99, *p*<0.05). Experiment 1 therefore confirmed that simulation richness is a crucial factor in the ability to favor delayed options when opposed to directly observable options.

Participants of the main MRI study (*n* = 20, Experiment 2) performed the same monetary and episodic intertemporal choice tasks. In both experiments, the observed choices were well predicted by the difference in discounted value between the two options (Figure 2C). We then examined whether behavioral performance was consistent across the episodic and monetary tasks, in order to establish that subjective ratings could be discounted similarly to classical payoffs. We found that impulsive choices were obtained with similar frequency in the monetary and episodic tasks (46.32%±8.26% and 50.90%±17.57% of impulsive choices, respectively). Moreover, nonimpulsive choice rate was significantly correlated across participants between the two tasks (Figure 2D, robust regression, t_32_ = 2.58, *p*<0.01), arguing in favor of a common underlying impulsivity trait. Consistently, the discount factor k was significantly correlated across individuals between monetary and episodic tasks (robust regression, t_32_ = 3.57, *p*<0.001). Thus, the form of impulsivity that is characterized by steeper discounting with delay was observed in the episodic as well as in the monetary task.

Focusing on the episodic task, we verified that the model fit was equally good in all conditions, to ensure that the imaging contrasts reported hereafter were valid. Prediction scores were calculated as the percentage of trials in which the option with the higher value estimate was chosen. Importantly, there was no significant difference in prediction scores between control and ecological trials (80.22%±1.31% and 78.47%±1.37%, paired *t* test: t_33_ = −1.26, *p*>0.2). The average difference in estimated values of chosen and nonchosen options was also very similar (control, 3.40±0.21; ecological, 3.46±0.20; paired *t* test: t_33_ = −0.28, *p*>0.3).

Overall, behavioral results validate our original episodic task, suggesting that participants weigh delays as they would do in classical economic paradigms (following hyperbolic discounting) but valuate delayed options in proportion to their simulation richness. Only the episodic task, not the monetary task, was used in the following analyses.

### Common Brain Activations for Valuations and Decisions

All activations reported below survived family-wise error (FWE) correction for multiple comparisons, either for the whole brain (noted WBC) or for a small volume (noted SVC) corresponding to anatomical delineation of the hippocampus (noted HC). We started with the identification, using GLM1 (see Methods), of brain regions encoding values and choices across participants.

**Figure 3:**
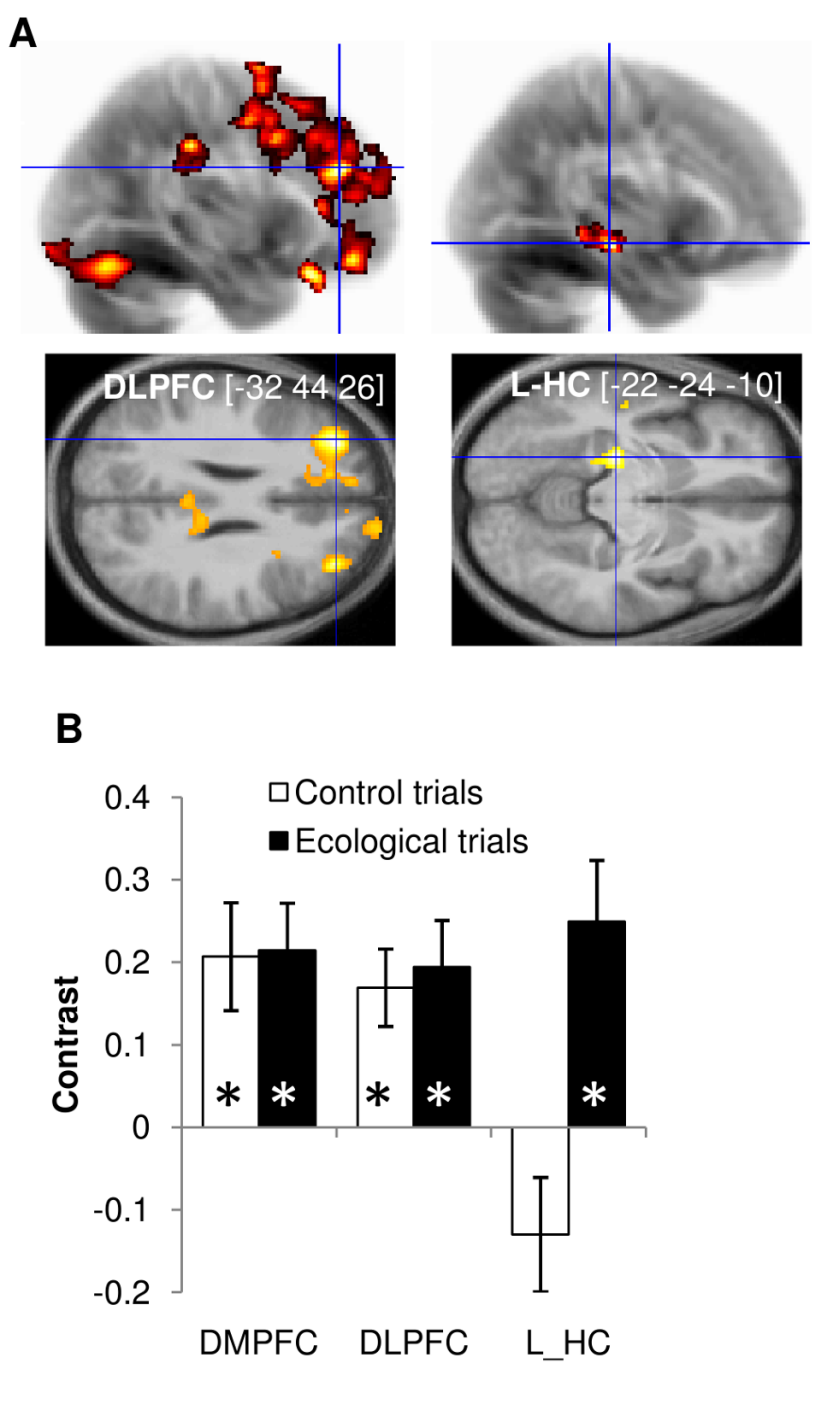
Group-level neural correlates of choices. (A) Statistical parametric maps. Left: contrast between nonimpulsive and impulsive choices including all trials, at the time of decision-making. Right: comparison of this same contrast between ecological versus control trials (i.e., Choice×Condition interaction). The color code on glass brains (top maps) and axial slices (bottom maps) indicates the statistical significance of clusters that survived the illustrative threshold (more than 200 voxels with *p*<0.005). The [x y z] coordinates of the different maxima refer to the Montreal Neurological Institute (MNI) space. Slices were taken in the different regions of interest (ROIs), along planes indicated by blue lines on glass brains. (B) Regression coefficients (betas). Bars indicate the contrast between nonimpulsive and impulsive choices for the ecological and control trials. The ROIs were defined as the intersection of functional activations and anatomical templates. Error bars indicate intersubject standard errors of the mean. HC, hippocampus; DLPFC, dorsolateral prefrontal cortex; DMPFC, dorsomedial prefontal cortex.

We first looked for brain regions that parametrically encode option values in the episodic task, collapsing all trial types (Figure S1). This brain valuation system encompassed numerous regions (all p_FWE_WBC_<0.05), such as the ventromedial prefrontal cortex (VMPFC), lateral parietal cortex (LPC), posterior cingulate cortex (PCC), and dorsolateral prefrontal cortex (DLPFC). We then analyzed the activity recorded during choice period, looking for regions that reflect choosing delayed options (nonimpulsive choices), regardless of trial type. A large prefrontal network, extending from the bilateral DLPFC to the dorsomedial prefrontal cortex (DMPFC), was significantly more activated (p_FWE_WBC_<0.05) for nonimpulsive than for impulsive choices (Figure 3A, left).

Next we searched for regions that would be specifically recruited for nonimpulsive choices in the ecological trials (Figure 3A, right). The interaction between choice and condition elicited specific activation in the left hippocampus (L_HC, p_FWE_SVC_<0.01; bi_HC p_FWE_SVC_ = 0.05). An ROI analysis (Figure 3B) confirmed the dissociation between prefrontal regions (DLPFC and DMPFC), which were more activated for nonimpulsive choices in both control and ecological trials, and left hippocampus, which was specifically engaged when subjects made nonimpulsive choices in the ecological trials (one-tailed paired *t* tests, *p*<0.05).

Thus, the analysis of brain activity suggests that the hippocampus is specifically involved in choosing delayed options when they need to be simulated, against immediate options that are directly observable (i.e., in Obs/Sim trials). This is in line with the hypothesis that hippocampus activity is proportional to simulation richness and therefore to the value of simulated options. This hypothesis predicts the observed absence of hippocampus activation in the two control conditions, for different reasons. In Obs/Obs trials, there is no need for simulation, and hence no need for hippocampus activation. In Sim/Sim trials, there are two options to simulate, but their value is on average the same for impulsive and nonimpulsive choices. This is why the contrast between impulsive and nonimpulsive choices yields no activation in the hippocampus.

### Correlation of Impulsivity with Brain Activity in Healthy Subjects

We explored interindividual differences, in order to provide additional evidence for the role of the hippocampus in resisting impulsive choices during ecological trials.

**Figure 4:**
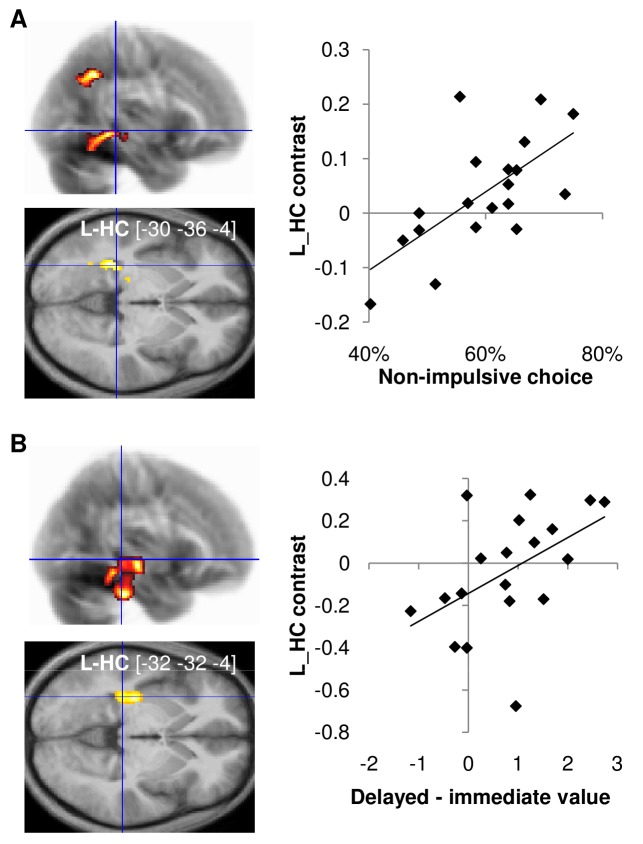
Neuro-functional correlates of inter-individual differences in choice impulsivity. (A) Correlations between neural contrast of nonimpulsive versus impulsive choice and behavioral nonimpulsive choice rate. (B) Correlation between neural valuation (contrast between activations elicited by delayed versus immediate option presentation) and behavioral valuation (difference between model-based estimates of delayed and immediate option values). Left: Statistical parametric maps, testing for higher correlation in ecological versus control trials. The color code indicates the statistical significance of clusters that survived the illustrative threshold (more than 200 voxels with *p*<0.005). The [x y z] coordinates of local maxima refer to the Montreal Neurological Institute (MNI) space. Right: Neuro-behavioral correlations in the ecological condition. The neural estimates were obtained from the intersection between clusters activated on the maps and an anatomical delineation of the hippocampus. Diamonds represent individuals; solid lines indicate robust regression fits. L-HC, left hippocampus.

We first took advantage of the intersubject variability in the rate of impulsive choices. Using GLM2 (see Methods), we specifically looked for regions in which the correlation between the nonimpulsive minus impulsive choice contrast and the nonimpulsive choice rate was higher in the ecological compared to the control condition (Figure 4A, left). This analysis, controlling for factors such as age, gender, and global correlation, revealed a significant cluster in the left hippocampus (L_HC, p_FWE_SVC_<0.05; bi_HC, p_FWE_SVC_ = 0.07). Post hoc analysis confirmed that the signal extracted from this L_HC ROI was positively correlated across subjects with nonimpulsive choice rate in the ecological condition (robust regression, t_17_ = 3.9, *p*<0.001, Figure 4A, right). Thus, subjects who exhibited less impulsivity in ecological choices had stronger hippocampus activation when choosing delayed options.

To provide further insight into the relationship between neural activity and behavioral impulsivity, we investigated correlations across individuals between the values inferred from the behavior and the activation measured at the time of option display (Figure 4B). More precisely, the two variables tested for these correlations were the difference in value estimates between delayed and immediate options (which we termed “behavioral valuation”) and the difference in activation between delayed and immediate options (which we termed “neural valuation”). We used GLM3 (see Methods) to search for regions showing higher correlation in ecological than in control conditions (Figure 4B, left). We found a significant cluster in the left hippocampus (L_HC, p_FWE_SVC_<0.01; bi_HC, p_FWE_SVC_<0.05). Thus, the individual propensity to value delayed options more than immediate options was linked with more activation in the hippocampus for delayed than for immediate option presentation. Post hoc analysis of the signal extracted from the L_HC ROI (Figure 4B, right) confirmed that intersubject correlation between behavioral and neural valuation was significantly positive in the ecological trials (robust regression, t_17_ = 2.72, *p*<0.05).

### Correlation of Impulsivity with Brain Anatomy in Healthy Subjects

We next examined whether interindividual differences in brain structure could account for the propensity to favor nonimpulsive options in our ecological condition. For this we performed VBM analysis (see Methods) on T1-weighted anatomical scans (*n* = 18). As a first step, we tested whether grey matter (GM) density in the L_HC (ROI from Figure 4A, top) could account for nonimpulsive choice rate. We found a significant correlation in the ecological trials (robust regression, t_16_ = 1.84, *p*<0.05) but not in the control trials (robust regression, t_16_ = 011, *p*>0.4). Similar results were obtained for the right or bilateral hippocampus. We also performed a whole-brain analysis that directly regressed nonimpulsive choice rate in the ecological condition against individual segmented GM maps, controlling for age, gender, and total intracranial volume (Figure S2). We found a small set of significant clusters, among which the hippocampus (R_HC, p_FWE_SVC_<0.05; bi_HC, p_FWE_SVC_ = 0.07).

**Figure 5:**
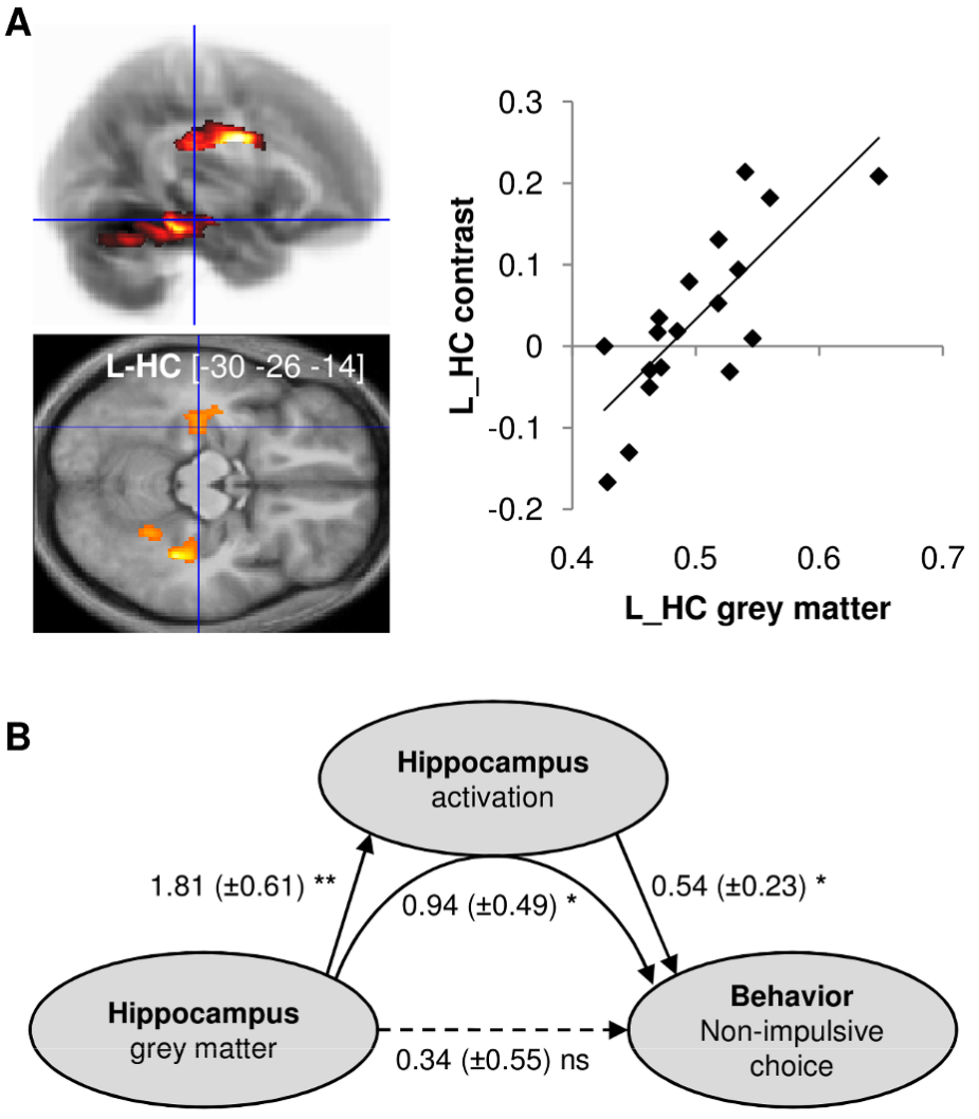
Neuro-anatomical correlates of interindividual differences in choice impulsivity. (A) Correlation between grey matter density and nonimpulsive versus impulsive choice contrast in the hippocampus ROI (cluster activated in Figure 4A). Left: Statistical parametric maps testing for higher correlation with the ecological versus control contrasts. The color code indicates the statistical significance of clusters that survived the illustrative threshold (more than 200 voxels with *p*<0.005). The [x y z] coordinates of local maxima refer to the Montreal Neurological Institute (MNI) space. Right: scatter plot for illustration of the anatomo-functional correlation in the ecological condition. Diamonds represent individuals; solid line indicates robust regression fit. ROI grey matter estimates were obtained from the intersection between the clusters activated on the map and an anatomical delineation of the hippocampus. L-HC, left hippocampus. (B) Mediation analysis of the links between anatomy, activity, and behavior. Grey matter density and functional activation were extracted from the cluster of Figure 4A, intersected with an anatomical delineation of the hippocampus. Solid and dashed arrows indicate significant and nonsignificant paths, respectively. Regression coefficients and standard errors (in brackets) are noted for each path. Statistical significance: * *p*<0.05; ** *p*<0.01; ns, non-significant.

We then examined whether the link from brain structure to behavioral choice could be mediated by brain activity. For this we extracted the nonimpulsive versus impulsive contrast from the L_HC ROI (Figure 4A, top), for both the ecological and control conditions. These functional contrasts were regressed against segmented GM maps, controlling for age, gender, and total intracranial volume. We then searched for regions were GM density was more correlated with functional contrast in the ecological than in the control condition. We again found a significant cluster (Figure 5A, left) in the hippocampus (L_HC, p_FWE_SVC_ = 0.059). Post hoc ROI analysis (Figure 5A, right) confirmed that intersubject correlation between GM density and functional activation was significantly positive in the ecological condition (robust regression, t_16_ = 4.64, *p*<0.001).

Thus, GM density in the left hippocampus accounted for both the individual propensity to favor delayed options in our ecological condition and the related hippocampus activation (nonimpulsive minus impulsive contrast in the ecological condition). A simple explanation of these statistical dependencies is that hippocampus activation mediates the relationship between GM density and behavioral choice (Figure 5B). To test this hypothesis, we performed a mediation analysis (see Methods). Results revealed that, when including hippocampus activation as a mediator, the direct path from anatomy to behavior was no longer significant. On the contrary, all the links of the indirect path (anatomy to activation to behavior) were significant (all *p*<0.05). One could argue that the VBM results may somehow confound the fMRI results, because a region with more neurons will show more activation, irrespective of functional implications. This is only true of course if those neurons are concerned with the contrast used to elicit functional activation, which we precisely intended to demonstrate. Furthermore, fMRI data established hippocampus functional implication in nonimpulsive choice not only in between-subject correlation but also in within-subject contrast, which cannot be driven by anatomical variations across participants.

To summarize, the functional and structural MRI data suggest that subjects with higher GM density in the hippocampus show more pronounced hippocampus activation in ecological trials and therefore better resistance to impulsivity. We note, however, that the statistical links demonstrated so far have no directionality. It could be argued that less impulsive subjects have higher hippocampal activation because they tend to simulate future options with more details. The same reasoning can apply to hippocampus anatomy, if we assume that activating a brain structure can increase its density. To eliminate the possibility that the anatomo-functional properties of the hippocampus are just a by-product of subjects liking future options, we investigated the consequence of hippocampal damage.

### Impulsivity Following Brain Atrophy in AD and bvFTD Patients

To assess whether intact hippocampus is necessary for preventing choice impulsivity, we compared the performance of AD patients to that of bvFTD patients and elderly controls in the episodic intertemporal choice task.

**Figure 6:**
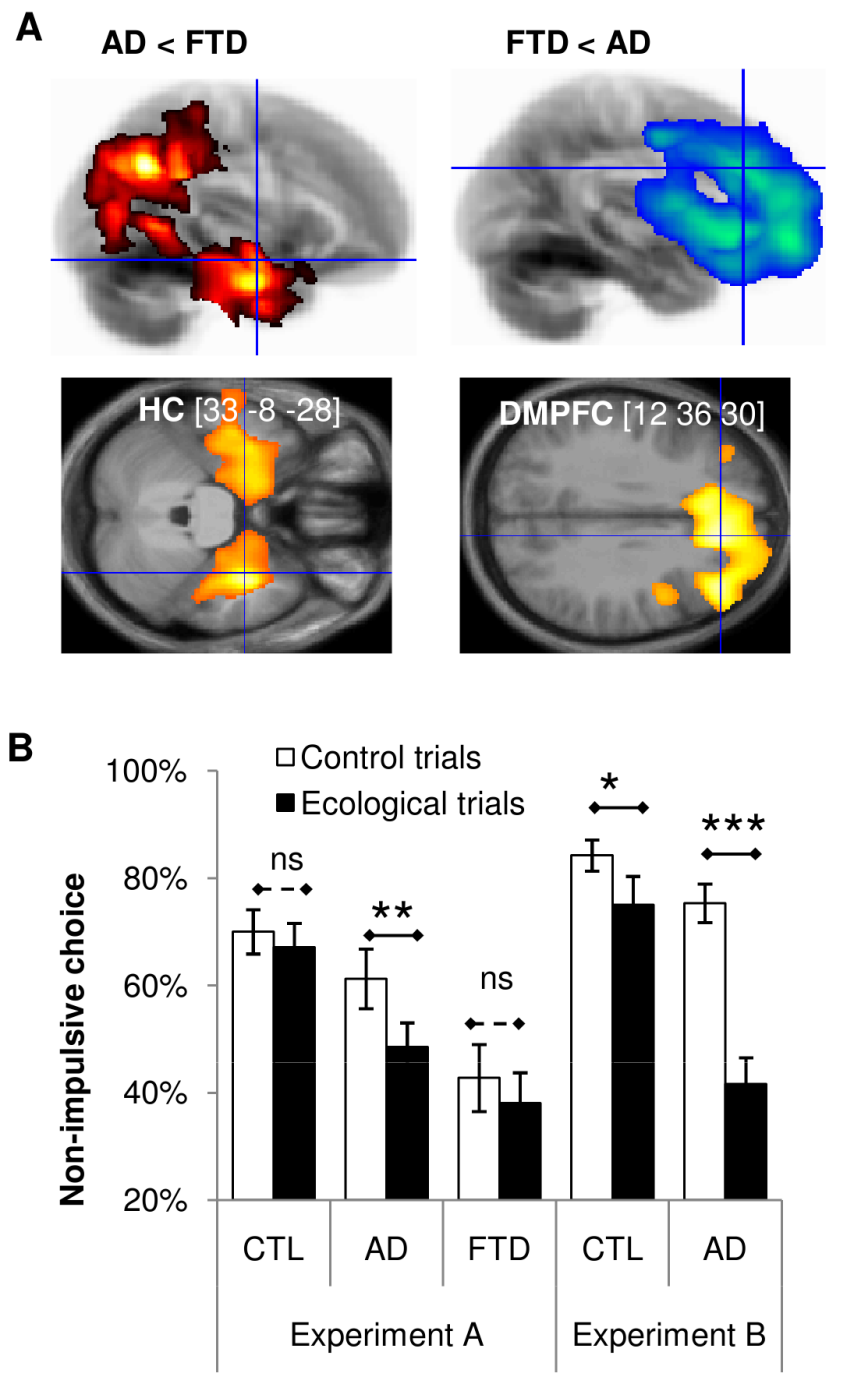
Brain structure and choice impulsivity in AD versus bvFTD patients. (A) Statistical parametric maps. The color code on the glass brains (top line) and axial slices (bottom line) indicates significant difference in grey matter density between AD and bvFTD patients (more than 10,000 voxels with *p*<0.05). Left: regions with reduced GM density in AD relative to bvFTD patients. Right: regions with reduced GM density in bvFTD relative to AD patients. The [x y z] coordinates of the global maximum refer to the Montreal Neurological Institute (MNI) space. (B) Intertemporal choices. Bars indicate nonimpulsive choice rate for the different groups and conditions in Experiments A (left) and B (right). Error bars indicate intersubject standard errors of the mean. AD, Alzheimer's disease; bvFTD, behavioral variant of fronto-temporal dementia; CTL, healthy control. Statistical comparison (two-sample *t* test): * *p*<0.05; ** *p*<0.01; *** *p*<0.001; ns, nonsignificant.

We first verified that AD and bvFTD patients recruited in the Pitié-Salpêtrière neurology wards (see Table 2 for demographic and clinical details) presented with a differential atrophy in the hippocampus. To this aim, we compared T1-weighted anatomical scans from 55 AD and 48 bvFTD patients using a two-sample *t* test on segmented GM maps, controlling for age, gender, mini-mental state (MMS), and total intracranial volume (see Methods). AD patients had reduced GM density in a large cluster (p_FWE_WBC_<0.05) that extended bilaterally from medial temporal regions to parietal lobules (Figure 6A, top), with a local maximum in the hippocampus (bi_HC, p_FWE_SVC_<0.05). Reciprocally, bvFTD patients had reduced GM density in a large prefrontal cluster (p_FWE_WBC_<0.05), mostly in the ventral and medial PFC areas (Figure 6A, bottom). This VBM analysis therefore confirmed that patients diagnosed with AD or bvFTD in our neurology wards were indeed characterized by specific neuro-degeneration patterns in temporo-parietal versus prefrontal regions, respectively.

**Table 2:** Characteristics of patients included in the VBM study.

Study	Group	Age (years)	Gender (% female)	MMSE (max 30)	FAB (max 18)	%1T (*n* = 21)	%1.5T (*n* = 61)	%3T (*n* = 21)
VBM study	AD (*n* = 55)	65.22 (±1.16)	0.52 (±0.07)	19.87 (±0.86)	12.58 (±0.63)	0.18 (±0.06)	0.64 (±0.07)	0.18 (±0.06)
	bvFTD (*n* = 48)	67.5 (±1.29)	0.50 (±0.07)	23.31 (±0.61)	12.12 (±0.70)	0.17 (±0.05)	0.67 (±0.07)	0.17 (±0.05)
*p* val	AD/FTD	0.18	0.78	<0.01	0.62	0.39	0.15	0.38

All cells contain the mean and its standard error (in brackets). The columns on the right indicate the proportion of patients for the different MRI scanners used to acquire T1 volumes. The *p* values in the bottom line correspond to the two-sample *t* tests comparing AD and bvFTD patients. AD, Alzheimer's disease; bvFTD, behavioral variant of Fronto-Temporal Dementia; FAB, Frontal Assessment Battery; MMSE, Mini-Mental State Examination.

We then administered the episodic intertemporal choice task (experiment A, see Methods) to 20 AD patients, 14 bvFTD patients, and 20 elderly controls (CTL) (see Table 3 for characteristics). AD and bvFTD patients were matched for global cognitive ability measured with Mini-Mental State examination (two-sample *t* test, t_33_ = 0.34, *p*>0.3). However, as expected, AD patients had more difficulty with episodic memory in the Free and Cued Selective Reminding Test (total free recall, two-sample *t* test, t_24_ = 2.47, *p*<0.01) and bvFTD patients with executive functions in the Frontal Assessment Battery (FAB score, two-sample *t* test, t_33_ = 2.70, *p*<0.01). Increasing delay significantly decreased the proportion of nonimpulsive choices in all groups (one-sample *t* tests; CTL, t_19_ = 6.43, *p*<0.001; AD, t_19_ = 5.90, *p*<0.001; bvFTD, t_14_ = 2.85, *p*<0.01). A first notable difference (Figure 6B, left) was that both groups of patients were on average more impulsive compared to healthy controls (two-sample *t* tests, AD versus CTL, t_38_ = 2.21, *p*<0.05; bvFTD versus CTL, t_33_ = 4.30, *p*<0.001; bvFTD versus AD, t_33_ = 2.29, *p*<0.05).

**Table 3:** Characteristics of patients included in the behavioral study.

Experiment	Group	Age (years)	Gender (% female)	Education level	MMSE (max 30)	FAB (max 18)	FCSRT (max 48)	Session completed
Experiment A	CTL	73.05 (±1.69)	0.5 (±0.11)	4.55 (±0.43)	28.95 (±0.18)	17.5 (±0.11)	NA (NA)	1.90 (±0.07)
	AD	76.25 (±1.62)	0.35 (±0.11)	5.25 (±0.36)	22.5 (±1.06)	14.3 (±0.42)	18.13 (±3.42)	1.90 (±0.07)
	bvFTD	65.57 (±2.39)	0.43 (±0.14)	4.93 (±0.56)	22.93 (±1.38)	11.43 (±0.89)	35.56 (±3.69)	1.93 (±0.06)
*p* values	AD/CTL	0.18	0.35	0.22	<0.001	<0.001	NA	—
	FTD/CTL	<0.05	0.69	0.59	<0.001	<0.001	NA	—
	AD/FTD	<0.001	0.65	0.62	0.80	<0.01	<0.01	—
Experiment B	CTL	71.27 (±1.74)	0.6 (±0.13)	4.73 (±0.42)	28.8 (±0.17)	17.47 (±0.13)	NA (NA)	1.93 (±0.07)
	AD	77.67 (±1.96)	0.53 (±0.13)	5.33 (±0.41)	22.27 (±0.54)	13.87 (±0.62)	19.33 (±3.75)	1.87 (±0.09)
*p* val	AD/CTL	<0.05	0.72	0.32	<0.001	<0.001	NA	—

All cells contain the mean and its standard error (in brackets). The column on the right indicates the average number of task sessions completed by the patients. The *p* values are from two-sample *t* tests. CTL, healthy controls; AD, Alzheimer's disease; bvFTD, behavioral variant of Fronto-Temporal Dementia; MMSE, Mini-Mental State Examination; FAB, Frontal Assessment Battery. FCSRT, Free and Cued Selective Reminding Test (total number of items retrieved during free recall).

The key test was the comparison of nonimpulsive choice rate between the ecological condition (Obs/Sim), in which the delayed option had to be mentally simulated, and the control condition (Obs/Obs), in which the delayed option could be visually observed. Unfortunately, the Group×Condition interaction tested with a global ANOVA did not reach the significance threshold (F_2,52_ = 12.52, *p* = 0.14). However, an exploratory analysis using *t* tests in each group separately fulfilled our predictions: AD patients were significantly more impulsive in the ecological condition (one-sample *t* test, AD, t_19_ = 2.61, *p*<0.001), not control subjects or bvFTD patients (one-sample *t* tests, CTL, t_19_ = 0.95, *p*>0.3; bvFTD, t_14_ = 0.97, *p*>0.1). Also, the difference between healthy controls and AD patients was significant for ecological but not for control trials (two-sample *t* tests, ecological, t_38_ = 2.84, *p*<0.001; control, t_38_ = 1.20, *p*>0.1), whereas the difference between healthy controls and bvFTD patients was significant in both conditions (two-sample *t* tests, ecological, t_33_ = 4.16, *p*<0.001; control, t_33_ = 4.02, *p*<0.001). Thus, pathological impulsivity was specifically revealed by the ecological condition in AD patients, but was exhibited irrespective of condition in bvFTD patients.

In order to confirm the specific deficit observed in AD patients, we modified the task by removing sport and culture options, which proved not suitable for aged and diseased subjects, and by shortening the delays, such that they were more adapted to elderly patients (Experiment B, see Methods). We recruited another 15 AD patients and 15 control subjects to try and replicate the results in an independent sample (Figure 6B, right). Crucially, the Group (AD versus CTL)×Condition (ecological versus control) interaction was this time significant (F_1,28_ = 12.52, *p*<0.01), Thus, AD patients made more impulsive choices than healthy controls, and this difference was driven by the ecological condition (two-sample *t* test, t_28_ = 5.10, *p*<0.001). Because the AD and CTL groups were not well matched in age, we verified that the group factor still explained the difference in impulsivity between ecological and control conditions when inserted into a GLM that also included a regressor for age. The GLM fit confirmed that group was a significant factor (t_28_ = 3.12, *p*<0.01) but not age (t_28_ = 0.57, *p*>0.5).

Thus, the behavior of AD patients suggests that damage to the hippocampus had an impact on intertemporal choices, since future options that needed mental simulation were no longer favored.

## Discussion

In this study, we extended standard delay discounting paradigms investigating intertemporal choices between smaller-sooner and bigger-later monetary payoffs. First, we developed an episodic choice task using more concrete options such as food, sport, and culture items. Second, we manipulated the mode of presentation to investigate ecological choices where the immediate option is directly observable, while the delayed option requires simulating a future episode. Behavioral data showed that richness of mental simulation is a crucial factor in valuating and hence choosing delayed options during ecological intertemporal conflicts. Imaging data revealed that interindividual variability in the propensity to favor simulated options can be explained by hippocampus functional activation during both valuation and choice, which in turn can be explained by the hippocampus grey matter density. Patient data demonstrated that AD, which is characterized by hippocampus atrophy, exacerbates impulsivity specifically when delayed options require mental simulation. Taken together, these results provided a strong support to our central hypothesis that the hippocampus helps valuating imagined outcomes, which reduces impulsivity in the context of ecological intertemporal choices [10]–[12]. In the following paragraphs we discuss behavioral data, functional MRI data, structural MRI data, and patient data, successively.

Behavioral data were modeled using a hyperbolic decay function to discount values with delays and a softmax rule to estimate choice likelihood. This model arguably provides a good account of intertemporal choices [4],[5],[34] and has become standard in the recent neuroeconomic literature [1],[2],[6],[28],[29]. We found that hyperbolic discounting provided an equally good fit of the monetary and episodic tasks—that is, whether we take objective financial payoffs or subjective likeability ratings as proxies for values. Note that because values were objective payoffs in one task and subjective ratings in the other, we could not assess whether episodic options attenuate discounting, as was shown previously [30],[31]. However, comparing with other discounting models would go beyond the scope of this study. Indeed, our focus was on how the brain assigns values to simulated options, not on how the brain discounts these values with delays. Importantly, the percentage of nonimpulsive choices, as well as the adjusted discount factor *k*, was correlated across subjects between the two tasks. This indicates that the same impulsivity trait, characterized as steepness of delay discounting, explains a significant part of variance in both financial and more concrete choices. Steepness of delay discounting might therefore be dissociated from the ability to simulate future episodes, which affected values irrespective of delays. Consistent with this idea, it was recently reported that a patient with impaired future simulation, due to hippocampal damage, exhibited normal discounting in a standard, monetary intertemporal choice task [35].

Imaging data corroborate previous findings [6],[7],[36], that nonimpulsive choices involved dorsal prefrontal regions (DLPFC and DMPFC), in both ecological and control trials. The novelty is the dissociation of hippocampus activation, which was specifically observed during ecological nonimpulsive decisions. Most hippocampus activations reported here were predominant on the left side, but survived small volume correction on both sides, when using bilateral masks of the hippocampus, independently defined from anatomical criteria. Thus, whereas the dorsal prefrontal cortex seems involved in preventing impulsivity during various types of choice, the hippocampus is specifically recruited for selecting simulated future options against directly observable options.

To our knowledge, this study is the first to implement the conflict between delayed options represented in episodic systems and immediate options represented in perceptual systems. Let us discuss the reasons why the hippocampus was activated by the contrast of nonimpulsive versus impulsive choice in this ecological situation, specifically. This contrast isolates choices where the simulated option was preferred and therefore imagined with greater detail, which according to our working hypothesis is underpinned by higher hippocampus activity. The same contrast did not activate the hippocampus in control conditions when the two options were observed, or when they were both simulated, for different reasons. When the two options can be represented in perceptual systems, there is no purpose for hippocampus activation since mental simulation is not required. When the two options are simulated they are presumably represented in episodic systems, but with similar richness irrespective of the choice, hence the absence of hippocampus activation when contrasting nonimpulsive and impulsive choices.

Although the idea that books elicit more imagination than movies seems well shared, it may be argued that pictures could also have yielded some simulation (for instance, imagining oneself consuming the food). Thus, observed and simulated options might not differ radically but rather in the degree of simulation needed for a proper valuation. We did not implement the contrast between immediate-simulated and delayed-observable options, because it would make little sense with respect to choice problems encountered in the real life. Indeed, immediately available options cannot be far from our senses, and our senses cannot directly perceive future events. We would nonetheless argue that it is the simulation process, and not the temporal frame (future against present), that determines the recruitment of hippocampus; otherwise, we would have observed hippocampus activation during nonimpulsive choices in the control conditions.

Yet we do not take position on which subprocess of mental simulation (such as retrieving pieces of information, reassembling these pieces into a new structure, representing the affective content of the simulation etc.) was implemented by the hippocampus. We also acknowledge that, due to practical constraints, our immediate-observable options were not truly obtainable at the moment of choice (but only after the fMRI session was over). The contrast with future-simulated options might have been even more powerful had subjects been confronted with the real object physically present at a reachable distance, either because it would have had more concreteness [12] or because it would have triggered Pavlovian consummatory processes [37]. Finally, let us emphasize that behavioral and fMRI data provide no indication about the direction of causality between valuation and simulation. The correlation observed in the behavior between simulation richness and likeability rating could reflect the fact that subjects imagined in more details what they liked in the first place. In this framework, variations of hippocampus activity could represent a by-product (and not a cause) of the valuation process. It was therefore crucial to assess the existence of a directional link from the simulation to the valuation process—that is, to demonstrate the necessity of hippocampus recruitment for valuating simulated options, which we did with patient studies.

To demonstrate that hippocampus-mediated simulation could explain some part of choice impulsivity, we explored interindividual variability. We found that the individual difference in value between delayed and immediate options (behavioral valuation) was correlated with the individual difference in activation between delayed and immediate options (neural valuation) in the hippocampus specifically during ecological trials. Thus, higher values assigned to delayed options correlated with both richer future simulation in behavioral data and stronger hippocampus activation in neuroimaging data. This is in line with the hypothesis that hippocampus-mediated future simulation helps valuating textually described options. This functional feature was corroborated by the anatomical observation that participants with higher grey matter density in the hippocampus have a higher propensity to make nonimpulsive choices in the ecological situation. Moreover, the relation between hippocampus anatomy and choice impulsivity was mediated by differential hippocampus activation in nonimpulsive versus impulsive choices. We therefore suggest that, on top of an impulsivity trait that relates to how delays are weighted and hence would affect any form of intertemporal choices, some individual variability in resisting observable rewards and favoring simulated options relies on the hippocampus structure and function. Again, the role of the hippocampus would not be to adjust the impact of delay but to provide a simulation that would make the delayed option more attractive.

One obvious clinical implication is that patients suffering from hippocampal damage, such as in AD, might encounter difficulties in pursuing long-term goals, due to deficient future simulation. To examine this possibility, we compared AD patients to patients with bvFTD, which is also a degenerative disease that progressively induces dementia in the aged person. Unfortunately, the overlap with the patients who performed our episodic intertemporal choice task was only partial, precluding direct correlations between neural degeneration and behavioral performance. Yet a direct comparison of brain anatomy between groups showed that GM density reduction preferentially affected prefrontal regions in bvFTD patients and MTL regions (including the hippocampus) as well as parietal areas in AD patients. This pattern could be expected from previous studies that compared AD and bVFTD groups to patients with mild cognitive impairment or to healthy controls [38]–[41]. Nevertheless, the clear-cut dissociation obtained here was not trivial, because of the common pathological features shared by the two pathological conditions [42]. This direct comparison between patient groups is certainly more stringent than the traditional comparison with healthy controls. Thus, we can reasonably assume that our AD patients had hippocampus degeneration, which validates our prediction that they should be particularly impulsive in the ecological condition. We note, however, that these patients also had other atrophic brain regions, particularly in the parietal cortex. Therefore, the patient study alone cannot be conclusive on hippocampus contribution to choice impulsivity. Nevertheless, the hippocampus appears as the most parsimonious candidate, given that it was also implicated in fMRI and VBM studies. Furthermore, results of the mediation analysis suggest that the impact of anatomical damage on choice impulsivity is mediated by the inability to activate the hippocampus during ecological conflicts.

The behavioral performance showed the following dissociation: AD patients exhibited pathological impulsivity in the ecological situation specifically, whereas bvFTD patients were found impulsive in all situations. The impulsivity observed in bvFTD patients accords well with the disinhibition syndrome that is classically reported in this variant of FTD [43]–[45]. It remains unclear whether their impulsivity emerges from a deficit in valuating options or in controlling choices. On the contrary, AD patients showed normal behavior in control conditions, ruling out any general impairment in valuation or choice. Instead, their impulsivity was revealed when no visual support was provided for the delayed option, which hence required simulation to be properly valuated. This enhanced impulsivity was driven by the food domain, which arguably proposes more tangible options than culture and sport domains, and hence a better contrast between immediately available rewards and simulated future events. The idea that AD patients are impulsive might seem counterintuitive to clinicians, as AD has rather been associated with apathy [46]–[48]. Apathy and impulsivity are not incompatible, however; indeed they frequently coexist in the same patients. We suggest that the inability to simulate future situations might also explain a lack of motivation in AD patients, precisely for long-term goals that cannot be visualized in their immediate environment. We replicated the demonstration of specific impulsivity during ecological intertemporal conflict in AD patients, using a short version of the task that can be administered in a few minutes. The test was therefore robust enough to overcome the fact that food preferences may vary across patients. This short test might prove useful in detecting motivational disorders in AD patients, and possibly in distinguishing AD from other degenerative diseases.

## Methods

This study was approved by the local Ethics Committee of the Pitié-Salpêtrière Hospital. All subjects and patients signed informed consent forms before performing tasks.

### MRI Study in Healthy Subjects

#### Subjects

All participants were screened for exclusion criteria: left-handedness, age under 18, regular usage of drugs or medication, history of psychiatric or neurological illness, and contraindications to MRI scanning (pregnancy, claustrophobia, metallic implants). They believed that they would be playing for a reward that would be randomly drawn from their choices (including both the monetary and episodic tasks) and given to them after the corresponding delay. To make this plausible, subjects could see in the lab a box full of food items and tickets for cultural and sport events. Eventually, the same immediate monetary reward (30€ in Experiment 1, 100€ in Experiment 2), and not any concrete option, was given to all subjects. Fifteen participants (5 females, age 24.90±2.31) were recruited via the “Laboratoire d'Economie Expérimentale de Paris” for Experiment 1 (behavioral pilot). Twenty different participants (8 females, age 23.65±0.87) were recruited via the “Relais d'Information sur les Sciences de la Cognition” website for Experiment 2 (fMRI study). One was excluded for always choosing the option displayed on the right side of the screen.

#### Behavioral tasks

All tasks were programmed on a PC, using the Cogent 2000 (Wellcome Department of Imaging Neuroscience, London) library of Matlab functions for stimuli presentation. We implemented two types of tasks: intertemporal choice tasks and likeability rating tasks.

In intertemporal choice tasks, subjects had to choose between two options: a less pleasant but immediate option versus a more pleasant but delayed (by 1 month, 1 year, or 10 years) option. We also manipulated the mode of presentation: observed (Obs) options were displayed as pictures accompanied with a short verbal description, whereas simulated (Sim) options were described with words only. We thus had three types of trials (Obs/Obs, Obs/Sim, and Sim/Sim), which we could divide into two conditions: the control condition (Obs/Obs and Sim/Sim trials) and the ecological condition (Obs/Sim trials). The ecological condition corresponds to everyday life situations where immediate rewards can be observed, while delayed ones have to be simulated.

Subjects underwent three sessions composed of 72 choice trials (36 Obs/Sim, 18 Obs/Obs, and 18 Sim/Sim trials, such that an equal number of Obs and Sim options were presented in the ecological and control conditions). Two sessions presented the “episodic” task, in which options were food, culture, or sport items (Figure 1A and Table 1). For every choice, the two options were pseudo-randomly drawn without replacement from the item list of a particular domain. The three factors (domain: food, culture, or sport; condition: ecological or control; and delay: 1 month, 1 year, or 10 years) were fully crossed, such that each delay and domain appeared with an equal frequency in the different conditions. The different combinations (cells) of the design were presented in a randomized order. The third session presented the “monetary” task, in which options proposed financial payoffs in euros (Figure 1B), and was performed between the two episodic sessions. Immediate options were randomly drawn without replacement in the [0.5 1 1.5 … 36] vector and delayed options were calculated as the immediate option plus an extra amount randomly drawn without replacement in the [1 2 3 … 36] vector. As in the episodic sessions, the condition and delay factors were fully crossed and the presentation order of the different combinations was randomized.

The trial structure was as follows. After a 0.5 s to 2.5 s fixation cross, the immediate and delayed options were presented sequentially, in a counterbalanced order, each for a duration that was jittered between 2 s and 5 s. Then, the verbal descriptions of the two options were displayed side by side, the left or right position of the immediate and delayed options being counterbalanced across trials. Subjects were asked to indicate which option they preferred by pressing the corresponding button, with their left versus right index finger. Choices were classified as “nonimpulsive” when subjects selected the delayed option and “impulsive” when they selected the immediate one.

Likeability rating tasks were administered after the intertemporal choice sessions. Subjects were asked to rate how much they liked each option of the episodic sessions, irrespective of delays. After a 1 s fixation cross, the item was presented during 1 s, with the same display as in the choice task (words plus picture or words only), with no mention of delay. Then appeared a scale graduated from −10 (not desirable) to 10 (highly desirable), with 1-point steps. Subjects had to move the cursor left or right by pressing the left or right arrow on the keyboard. Finally they pressed the space key to validate their response and to proceed to the next trial.

In Experiment 1, an additional rating task was implemented to assess simulation richness. Subjects were asked to indicate how many details they evoked when reading each future option of the episodic sessions, irrespective of delays. Trial structure was identical to the likeability rating task except that the scale was graduated from 0 (no detail at all) to 20 (maximum possible).

#### Computational model

Likeability ratings (for the episodic condition) and financial amounts (for the monetary condition) were used as value proxies for the different items. Financial amounts were transformed such that they were distributed over the same [−10, 10] interval as likeability ratings. These values were hyperbolically discounted with the delay: V = R/(1+kD), where R is the likeability rating or monetary reward, D is the delay in days, and k the discount rate. Given the discounted values (V), the probability (likelihood) of selecting each option was estimated using a softmax rule—for example, for an impulsive choice: Pimp = exp(Vi/β)/{exp(Vi/β) + exp(Vd/β)}, where β is the temperature, Vi the value of the immediate option, and Vd the discounted value of the delayed option. The free parameters (k, β) were individually adjusted over all conditions and domains to maximize the log likelihood of the actual choices under the model. For fMRI analyses of the episodic task, a single set of parameters was used for all subjects, such that estimations were based on a more comprehensive data set.

#### MRI data acquisition

T2*-weighted echo planar images (EPI) were acquired with blood oxygen-level dependent (BOLD) contrast on a 3.0 Tesla magnetic resonance scanner (Siemens Trio). We employed a tilted plane acquisition sequence designed to optimize functional sensitivity in the orbitofrontal cortex and medial temporal lobes [49],[50], with the following parameters: TR = 2.0 s, 35 slices, 2 mm slice thickness, 1.5 mm interslice gap. T1-weighted structural images were acquired (1 mm isotropic, 176 slices), co-registered with the mean EPI, segmented and normalized to a standard T1 template, and averaged across subjects to allow group-level anatomical localization. Imaging data were preprocessed and analyzed using SPM8 (Wellcome Trust Center for NeuroImaging, London, UK) implemented in Matlab. The first five volumes of each session were discarded to allow for T1 equilibration effects. Preprocessing consisted of spatial realignment, normalization using the transformation computed for the segmentation of structural images, and spatial smoothing using a Gaussian kernel with a full-width at half-maximum (FWHM) of 8 mm.

#### MRI data analysis

Functional images acquired during the episodic task were analyzed in an event-related manner, using three general linear models (GLMs) to explain individual-level functional scans. GLMs 1 and 3 modeled three events per trial (immediate and delayed option presentations, plus the choice period), with boxcar functions. Because there were two types of trials (control and ecological), the two GLM included six categorical regressors. GLM2 modeled one event per trial with a boxcar encompassing option presentations and choice period, for a total of two categorical regressors (for ecological and control trials). In GLM1, the regressors modeling option presentation were parametrically modulated by the subjective value (which was equivalent to likeability rating for immediate options but not for delayed options). The regressors modeling choices were modulated by three parameters: an indicator function for nonimpulsive versus impulsive choice (see above), an indicator function for the side of the immediate option display on the screen (1 for left and 0 for right), and the response time (RT). In GLM2, the two regressors were modulated by an indicator function for nonimpulsive versus impulsive choice. GLM3 was identical to GLM1 except that it did not include any parametric modulator. All regressors of interest were convolved with a canonical hemodynamic response function (HRF) and its first temporal derivative. To correct for motion artifacts, subject-specific realignment parameters were modeled as covariates of no interest.

Linear contrasts of regression coefficients (betas) were computed at the subject level, smoothed with 6-mm FWHM Gaussian kernel, and taken to a group-level random effect analysis, using one-sample *t* tests. Only the betas obtained for the canonical HRF were analyzed. Intersubject regressions were tested using second-level GLMs that included subject-specific contrast images and behavioral variables of interest. Three additional regressors were included in these GLMs to account for noninterest variance in age, gender, and global correlation (over the entire brain).

To test at the whole brain level that intersubject correlation was significantly higher in ecological than in control conditions, we used an approach similar to the one used in Psycho-Physiological Interactions (PPI). We built second-level GLMs to explain the dependent variable Y (individual images for the two conditions). These GLMs included one regressor X containing individual behavior (e.g., choice rate) for both conditions, a dummy variable Z indicating the condition, an interaction term X*Z, and the covariates of noninterest (gender, age, and global correlation over the entire brain). To test for a difference in correlation between conditions, we simply tested the significance of the regressor modeling interaction term.

All activation maps are displayed for illustration at a threshold of *p*<0.005 at the voxel level, and a number of contiguous voxels of 200. All activations reported in the main text survived either a family-wise error (FWE) correction for multiple comparisons over the whole brain at the cluster level (FWE_WB_), or a FWE small-volume correction within anatomical mask of the hippocampus (FWE_SVC_).

#### VBM

Preprocessing for VBM analysis [51] was carried out using the DARTEL [52] toolbox for SPM8 (http://www.fil.ion.ucl.ac.uk/spm). T1-weighted structural images were segmented into six classes of tissues in native space, which resulted in roughly aligned (through rigid transformation) grey and white matter (GM and WM) images. Both GM and WM images were then warped to an iteratively improved template using nonlinear registration in DARTEL. The final DARTEL template was affinely registered to the MNI space, and the individual GM images were wrapped using the DARTEL flow-fields and the last template affine transformation, in a way that preserved their local tissue volumes (equivalent to a Jacobian “modulation” step). GM maps were smoothed using a Gaussian kernel with 8-mm FWHM. We then ran a second-level GLM, including subject-specific modulated GM maps and nonimpulsive choice rates. Additional covariates of noninterest included gender, age, and total intracranial volume (TIV). T1 images were missing for one subject due to technical issues during acquisition.

#### Regions of interest (ROIs)

All ROI analyses were performed on the intersection of the significant cluster in the contrast of interest and an anatomical mask of the region of interest (e.g., hippocampus, DLPFC). As activation maps were produced by testing differences between conditions (ecological trials – control trials), the ROI analyses were used as post hoc confirmation that differences were driven by the condition of interest (i.e., by a positive effect in ecological trials). We nonetheless verified that the results hold when defining ROI based on purely anatomical criteria. Anatomical masks were based on the AAL brain parcellation [53] and created using MARINA software (http://www.bion.de/index.php?title=Home&lang=eng). The signal (functional contrast or GM density) was averaged over the whole ROI for each subject before statistical testing. Statistical significance was assessed either at the group level using one-sided one-sample *t* tests, or for intersubject correlations using one-sided *t* test on the studentized coefficient returned by the robust regression tool implemented in Matlab.

The mediation analysis between GM density, functional contrast, and choice impulsivity was implemented using the 8.14.2012 Mediation Toolbox (available at http://wagerlab.colorado.edu/tools). In brief, this mediation analysis tested whether the relationship between GM density (GM) and choice impulsivity (CHOICE) can be explained by the functional contrast (BOLD). This is the case if the direct path from GM to CHOICE (β_0_) is no longer significant when introducing the mediator BOLD, while the indirect path from GM to CHOICE through BOLD (β_1_×β_2_) is significant. Thus, the mediation analysis can be reduced to estimating the two following linear models: BOLD = β_1_×GM and CHOICE = β_0_×GM+β_2_×BOLD. Path significance was then assessed with a bootstrap test using 10,000 bootsamples.

### Behavioral Study in Patients with Brain Atrophy

#### Patients

Patients were sampled from the Neuroradiology Department and the Institute for Memory and Alzheimer's Disease at the Pitié-Salpêtrière Hospital. They were diagnosed based on neurological interview, neuro-psychological battery, psychiatric assessment, and MRI examination. All patients were in a predemented state, with a Mini-Mental State Examination (MMSE) score around 23/30 on average. Exclusion criteria were (1) clinical or neuroimaging evidence for focal lesions and (2) medical conditions that would interfere with cognitive performance.

AD patients fulfilled the National Institute of Neurological and Communicative Disorders and Stroke and the Alzheimer's disease and Related Disorders Association (NINCDS-ADRDA) criteria [54] for probable AD. Their memory impairment was characterized by the Free and Cued Selective Reminding Test (FCSRT [55]), as a low free recall performance (group average below 19/48) that was not compensated for by semantic cueing.

bvFTD patients fulfilled the revised Lund-Manchester consensus criteria for frontotemporal dementia [56],[57]. They presented with a corroborated history of initial progressive decline in social interpersonal conduct and behavior with emotional blunting and loss of insight. Patients with language disorders (progressive nonfluent aphasia or semantic dementia) were excluded. The dysexecutive syndrome of bvFTD patients was evidenced by low scores (group average below 12/18) on the Frontal Assessment Battery (FAB [58]).

Elderly control subjects were recruited at the Institut du Cerveau et de la Moelle epiniere (ICM). They had no history of neurological or psychiatric disorders. They did not complain about cognitive decline and did not take medications, such as antidepressants, anxiolytics, or neuroleptics. Individuals who scored lower than 27/30 in the MMSE or lower than 16/18 in the FAB were not included.

Unfortunately, there was a poor overlap between patients who performed our intertemporal choice task and patients for which MRI scan was available. We therefore conducted the VBM and behavioral studies in separate groups (see demographic and clinical details in Tables 1 and 2). The VBM study included 103 patients (55 AD and 48 bvFTD) and the behavioral study 84 participants (20 AD, 14 bvFTD and 20 healthy controls for Experiment A; another 15 AD and 15 controls for Experiment B). Patients and elderly controls were not paid for their participation.

#### MRI data acquisition and analysis

All images were T1-weighted anatomical whole-brain scans recorded in the neuroradiology department of the hospital using three different MRI scanners at 1T (Panorama Philips), 1.5T (Sigma 1.5T G.E Medical systems), and 3T (Sigma 3T HDX G.E Medical systems). Various sequences were used for an acquisition of 116 to 240 slices, with an interpolated thickness of the following range: [0.488 to 1]* [0.488 to 1]* [0.7 to 1.5] mm. Importantly, the repartition of the different scanners and sequences was equivalent for AD and bvFTD groups (see Table 1).

VBM preprocessing was identical to that used in healthy subjects, with 12-mm FWHM for spatial smoothing. Groups were compared with a second-level two-sample *t* test on subject-specific modulated GM maps. Additional covariates of noninterest included gender, age, total intracranial volume (TIV), and two dummy regressors to account for differences in scanner and sequence. A covariate for MMSE score was also added separately for each group, using the interaction option of SPM8.

#### Behavioral tasks

For Experiment A we used the exact same items and delays as for the MRI study in healthy subjects (Experiments 1 and 2), but the task was made shorter by skipping the successive option display periods, thus presenting choices only. Because numerous patients reported that sport and culture choices seemed awkward given their medical condition, we focused our analysis on food items. Also, the delay of 10 years that was initially used in young healthy subjects was not adapted for elderly diseased subjects. We therefore adapted the task for Experiment B, presenting only items of the food domain and reducing the longest delay to 5 years (the two others remaining 1 day and 1 month).

## Supporting Information

Figure S1 **Group-level neural correlates of values.** Statistical parametric maps show correlation with subjective values estimated by hyperbolically discounting likeability ratings with delays, at the time of option valuation. The color code on glass brains (left column) and slices (right column) indicates the statistical significance of clusters that survived the threshold (more than 200 voxels with *p*<0.005). The [x y z] coordinates of local maxima refer to the Montreal Neurological Institute (MNI) space. Slices were taken in local maxima of interest, along planes indicated by blue lines on glass brains. VMPFC, Ventromedial Prefrontal Cortex.
(TIFF)

Figure S2 **Anatomical correlates of interindividual differences in choice impulsivity.** Statistical parametric maps show correlation between grey matter density and nonimpulsive choice rate. The color code on glass brains (left column) and slices (right column) indicates the statistical significance of clusters that survived the threshold (more than 200 voxels with *p*<0.005). The [x y z] coordinates of local maxima refer to the Montreal Neurological Institute (MNI) space. Slices were taken in local maxima of interest, along planes indicated by blue lines on glass brains. R-HC, right hippocampus.
(TIFF)

## Abbreviations

AD: Alzheimer's disease
ANOVA: analysis of variance
BOLD: blood oxygen level dependent
bvFTD: behavioral variant of fronto-temporal dementia
CTL: control
DLPFC: dorsolateral prefrontal cortex
DMPFC: dorsomedial prefrontal cortex
EPI: echo-planar image
FAB: frontal assessment battery
FCSRT: free and cued selective reminding test
FWE: family-wise error
FWHM: full width at half maximum
GLM: general linear model
GM: grey matter
HC: hippocampus cortex
HRF: hemodynamic response function
LPC: lateral parietal cortex
MMSE: mini mental state examination
MRI: magnetic resonance imaging
MTL: medial temporal lobe
PCC: posterior cingulate cortex
ROI: region of interest
SEM: standard error of the mean
SPM: statistical parametric mapping
SVC: small volume correction
TIV: total intracranial volume
TR: repeat time
VBM: voxel-based morphometry
VMPFC: ventromedial prefrontal cortex
WBC: whole brain correction
WM: white matter
