# Supplementary figures and images for: A Critical Role for the Hippocampus in the Valuation of Imagined Outcomes
Source: PLoS Biol. 2013 Oct 22;11(10):e1001684. doi: 10.1371/journal.pbio.1001684 (PMC3805472; doi:10.1371/journal.pbio.1001684)

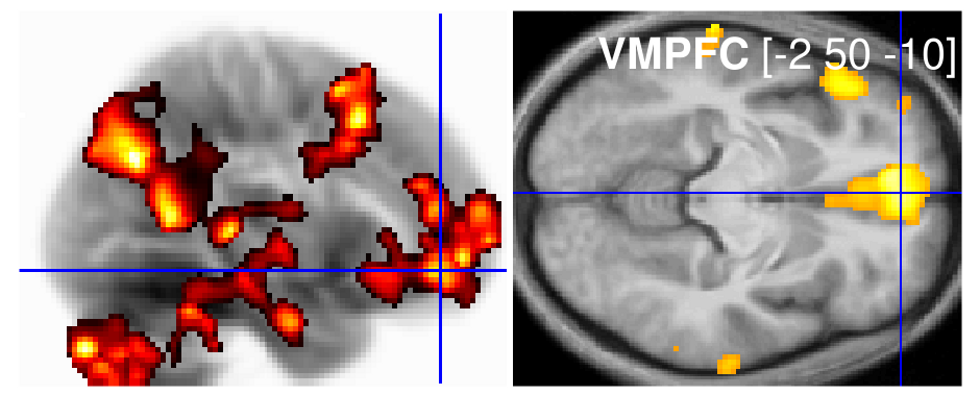

Supplement: Figure S1 — Group-level neural correlates of values. Statistical parametric maps show correlation with subjective values estimated by hyperbolically discounting likeability ratings with delays, at the time of option valuation. The color code on glass brains (left column) and slices (right column) indicates the statistical significance of clusters that survived the threshold (more than 200 voxels with p<0.005). The [x y z] coordinates of local maxima refer to the Montreal Neurological Institute (MNI) space. Slices were taken in local maxima of interest, along planes indicated by blue lines on glass brains. VMPFC, Ventromedial Prefrontal Cortex. (TIFF) [file pbio.1001684.s001.tiff]

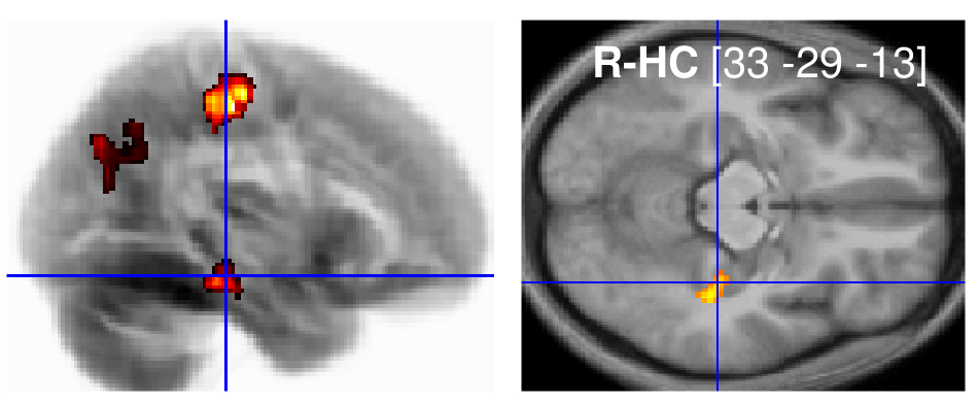

Supplement: Figure S2 — Anatomical correlates of interindividual differences in choice impulsivity. Statistical parametric maps show correlation between grey matter density and nonimpulsive choice rate. The color code on glass brains (left column) and slices (right column) indicates the statistical significance of clusters that survived the threshold (more than 200 voxels with p<0.005). The [x y z] coordinates of local maxima refer to the Montreal Neurological Institute (MNI) space. Slices were taken in local maxima of interest, along planes indicated by blue lines on glass brains. R-HC, right hippocampus. (TIFF) [file pbio.1001684.s002.tiff]
